# Impact of resistance and endurance exercise training on femoral artery function: sex differences in humans

**DOI:** 10.1113/JP287534

**Published:** 2025-02-06

**Authors:** Daniel J. Green, Hannah J. Thomas, Channa E. Marsh, Leanne Lester, Louise H. Naylor, Andrew Haynes

**Affiliations:** ^1^ School of Human Sciences (Exercise and Sport Science) The University of Western Australia Perth Western Australia Australia; ^2^ Business School (Centre for Social Impact) The University of Western Australia Perth Western Australia Australia

**Keywords:** arterial function, endothelium, exercise, prevention, sex differences

## Abstract

**Abstract:**

Exercise has direct and indirect anti‐atherogenic impacts on arterial function and health in humans. Few studies have directly compared the impacts of different commonly adopted exercise approaches on femoral artery function. We hypothesized that, owing to its direct impact via sustained increases in shear stress, endurance (END) training would have larger impacts on arterial diameter and function than resistance (RES) training. Thirty‐nine young, healthy participants (age 26.9 ± 6.2 years, 22♀) completed 12 weeks of both RES and END training in random order, separated by a 12 week washout. Resting femoral artery diameter and flow‐mediated dilatation (FMD) were collected before and after each exercise intervention. END training was associated with an increase in both FMD (Δ1.61 ± 3.09%, *P = *0.005) and resting diameter (Δ0.15 ± 0.29 mm, *P = *0.004). Neither resting diameter nor FMD increased following RES. However, sex difference analysis revealed that males increased FMD following RES (Δ2.21 ± 3.76%, *P = *0.015), whereas no RES change was evident for females. Following END, both males and females increased FMD (♂, Δ1.11 ± 1.65%; ♀, Δ1.88 ± 3.67%; both *P = *0.025), with males also showing an increase in resting arterial diameter following END (Δ0.23 ± 0.2 mm, *P* < 0.001). Group data revealed that END has greater impacts than RES on femoral artery diameter and flow‐mediated functional responses, which are endothelium mediated and nitric oxide dependent. Males exhibit beneficial impacts in response to both END and RES, whereas females respond predominantly to END. Our findings suggest that arterial adaptation to exercise might be influenced by exercise modality and sex.

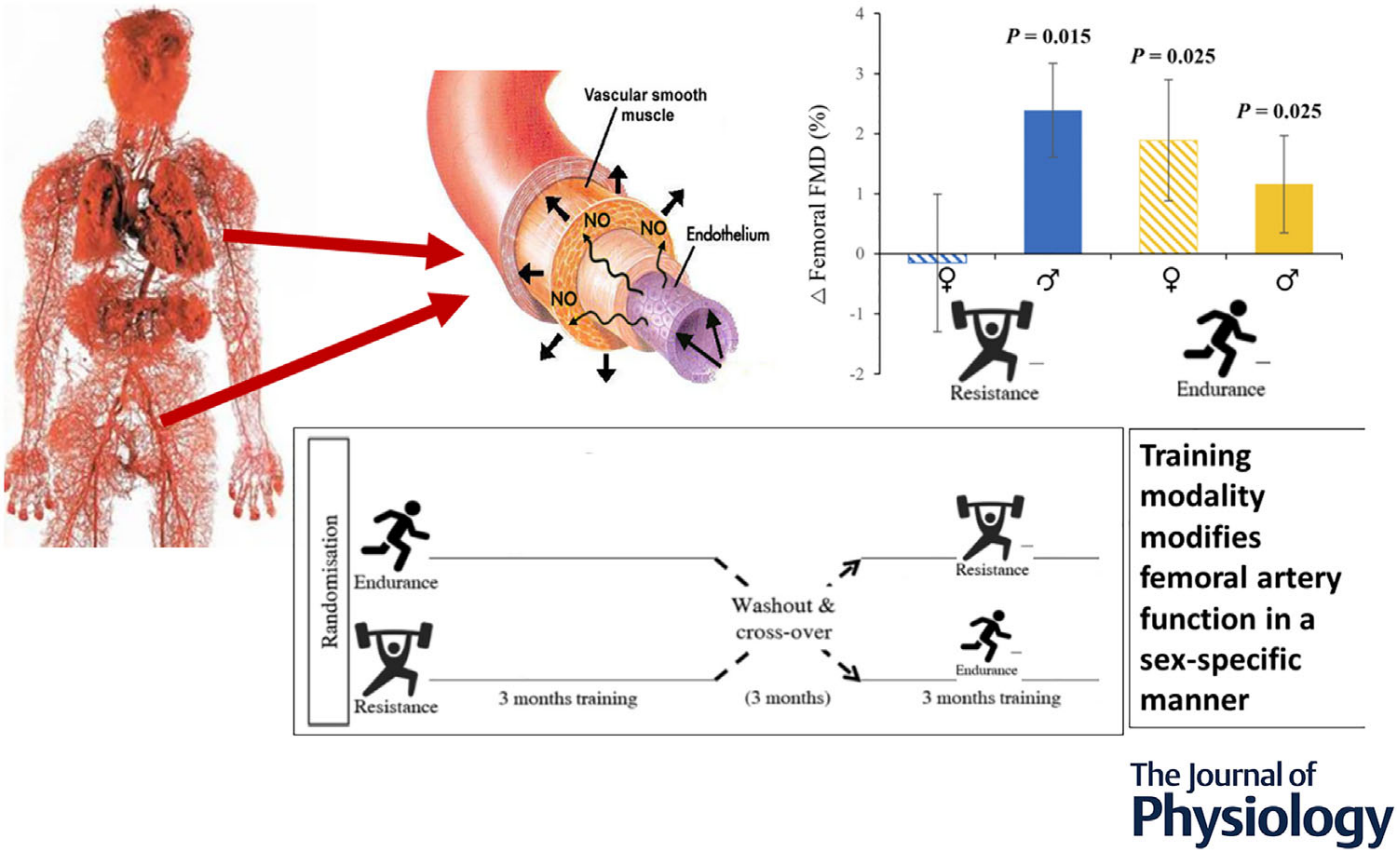

**Key points:**

- Exercise has anti‐atherogenic effects and lowers the risk of cardiovascular diseases. This is mediated, in part, by the direct haemodynamic impacts of exercise on arterial function, structure and health. Different modalities of exercise have distinct effects on arterial haemodynamics, but few studies have directly compared, within subjects and using a cross‐over design of trial, the relative impacts of distinct forms of exercise training on arterial adaptation.
- In this study, endurance training increased baseline femoral artery diameter and flow‐mediated dilatation, which is endothelium dependent and mediated by nitric oxide. Resistance training had a beneficial but lesser impact.
- Females and males were responsive to endurance training, but only males responded positively to resistance training in this study.
- These results show that changing the training mode modifies training‐induced arterial adaptation; this has implications for the optimization of exercise prescription for individual benefit.

## Graphical abstract

**Abstract figure legend** This randomized cross‐over study addressed the hypothesis that endurance training would have larger impacts on arterial diameter and function than resistance training. Endurance training increased flow‐mediated dilatation (FMD) and resting diameter, whereas resistance training did not modify either. However, sex differences were apparent. Males exhibited beneficial impacts in response to both endurance and resistance training, whereas females responded predominantly to endurance training. Our findings suggest that arterial adaptation to exercise might be influenced by exercise modality and sex.

## Introduction

Exercise enhances vascular endothelial function, including the bioavailability of nitric oxide (NO) and other paracrine hormones (Green et al., 2017; Green, O'Driscoll, Blanksby, et al., 1996; Hambrecht et al., 2003). This improvement might be a ‘secondary’ consequence of the impacts of exercise on traditional cardiovascular (CV) risk factors, such as blood pressure and lipid levels, or directly related to the episodic effects of exercise on arterial wall haemodynamics (Green et al., 2008). In previous experiments, we demonstrated that changes in arterial shear stress contribute to the beneficial impacts of exercise on arterial function and structure and that repeated increases in shear in the absence of exercise induce changes in arterial function and structure that emulate effects of exercise (Birk et al., 2012; Carter et al., 2014; Green et al., 2017; Naylor et al., 2011; Tinken et al., 2010).

Repeated dynamic contractions of large muscle groups that can be maintained for relatively long durations underpin exercise modalities often referred to as ‘endurance’ (END) exercises. Common modes of END, such as walking, running and cycling, persistently elevate blood pressure and blood flow to the working muscle throughout the exercise bout. Somewhat in contrast, muscle contractions performed against a high external load for relatively short durations are referred to as ‘weight‐training’ or resistance (RES) exercises. Participation in RES exercise is associated with phasic changes in blood pressure and localized muscle blood flows. Given that the modality of exercise performed has differential impacts on arterial haemodynamics and shear stress (Green et al., 2017; Spence et al., 2013; Thijssen et al., 2009; Tinken et al., 2009), arterial adaptation to training might be dependent upon the mode of exercise performed and the vessel bed under examination.

No previous study has directly compared the effects of END and RES exercise training on lower‐limb arterial function in a repeated‐measures cross‐over design study. We hypothesized that END training would have a larger impact than RES training on lower‐limb arterial function, given the sustained increase in arterial shear stress associated with END exercise. We also hypothesized that females would exhibit larger functional improvements than males.

## Methods

### Ethical approval

Full details of the study design can be found in our protocol paper (Marsh et al., 2020a). Study registration with the Australian New Zealand Clinical Trials Registry (ACTRN12616001095459) was completed prior to randomization, and the study was approved by the University of Western Australia Human Research Ethics Committee (reference RA/4/7031). Written informed consent was obtained from each participant prior to enrolment in the study, which conformed to the *Declaration of Helsinki*.

### Participants

Data presented in this paper relate to a cross‐over exercise intervention study of twin pairs, referred to as the STRUETH study (Studies of Twin Responses to Understand Exercise Therapy), involving 70 healthy but relatively inactive young adult participants (i.e. 35 twin pairs) (see Fig. S1). All individuals completed brachial vascular measurements, data from which have been published previously (Green et al., 2023). Femoral vascular data were collected in a subset of these participants, with data from 39 individuals (17 males and 22 females) included in this paper. This femoral measurement group was a convenience sample from the larger study, based on the availability of trained sonographers to perform femoral vascular measurements whilst the brachial measures were collected simultaneously by another experienced sonographer, along with the tolerance of participants to additional testing.

Participants were aged 26.9 ± 6.2 years, non‐smokers, self‐reported no cardiovascular or other diseases, and did not meet the Australian guidelines for physical activity of 150 min per week. Owing to the lower number of participants who had femoral artery scans performed, heritability analyses have not been included in this manuscript.

We consider that performing upper‐ and lower‐limb vascular measures simultaneously is a valid experimental approach, because it controls for any time‐related differences in subject status, haemodynamics and presentation. Baseline characteristics of the participants are presented in Table 1.

**Table 1:** Baseline characteristics of participants

Variable	All (*n* = 39)	Female (*n* = 22)	Male (*n* = 17)	Female *vs*. male *P*‐value
Age (years)	26.9 (6.2)	24.8 (4.5)	29.7 (7.0)	0.017
Height (cm)	173.7 (7.7)	168.1 (4.3)	180.8 (4.3)	<0.001
Weight (kg)	72.0 (16.7)	61.8 (9.7)	85.3 (14.2)	<0.001
BMI (kg m^−2^)	23.6 (3.9)	21.8 (3.2)	26.0 (3.6)	0.001
Waist:hip ratio	0.76 (0.08)	0.71 (0.04)	0.83 (0.05)	<0.001
Systolic BP (mmHg)	113 (9)	110 (5)	116 (12)	0.077
Diastolic BP (mmHg)	64 (8)	63 (6)	66 (11)	0.320
${\dot{V}}_{O_{2}}$ (ml kg^−1^ min^−1^)	39.6 (5.9)	36.9 (3.3)	43.1 (6.6)	<0.001
1RM bench press (kg)	46.0 (18.7)	31.7 (5.1)	64.4 (12.5)	<0.001
1RM leg press (kg)	174.5 (57.8)	135.8 (22.1)	224.5 (50.8)	<0.001
1RM bench press/weight	0.6 (0.2)	0.5 (0.1)	0.8 (0.2)	<0.001
1RM leg press/weight	2.5 (0.6)	2.3 (0.5)	2.8 (0.6)	<0.001

Data are the mean (SD). Abbreviations: BMI, body mass index; BP, blood pressure; 1RM, 1 repetition maximum; 1RM bench and leg press/weight, strength relative to body weight; ${\dot{V}}_{O_{2}}$, oxygen uptake. Female *vs*. male *P*‐values are from Student's *t* test.

### Study design

Participants were randomized to undertake either the 12 week RES or the 12 week END training intervention. Following their initial training intervention, each participant underwent a 12 week washout period, during which they were instructed to maintain their usual level of physical activity and usual diet. Participants then crossed over to complete their second, alternative, 12 week exercise intervention (RES or END). Physical activity was monitored (Actiheart 4; CamNtech Ltd, Cambridge, UK) to assess total and active energy expenditure in the week before or immediately after each exercise intervention to quantify incidental changes in physical activity levels. Participants wore the monitor for a 5 day period. A food diary was also given to each participant to complete the week before, or immediately after, each exercise intervention. Participants were instructed to complete the diary on 4 days consecutively, including one weekend day. The mean total daily energy intake was determined from these records using a commercially available software program (Foodworks 9; Xyris Software, Brisbane, QLD, Australia).

### Exercise interventions

Participants trained at matched relative intensities, with heart rate monitored continuously throughout the END exercise sessions (Polar RS300X HR monitor, Electro Oy, Finland). A detailed description of the exercise interventions is provided in our protocol paper (Marsh et al., 2020a). Briefly, each intervention (RES and END) consisted of three sessions per week for 12 weeks, which progressed to reach a 1 h session. The programmes were centre based, progressively overloaded and supervised by an Accredited Exercise Physiologist. END training consisted of two running and one cycling session per week, progressing from 60 to 90% maximum heart rate (based on individual peak oxygen uptake test data). RES sessions alternated between upper‐body (i.e. bench press, standing military press) and lower‐body (i.e. barbell back squats, deadlifts, leg press) exercises, progressing from 60 to 90% of one‐repetition maximum (1RM), with repetitions decreasing from 15 to 5 repetitions as load increased.

### Outcome measures

For the vascular testing sessions, participants were tested at the same time of the morning for repeat visits, in the same conditions (fasted; no alcohol and no moderate/vigorous physical activity for 24 h prior) (Thijssen et al., 2011). All vascular testing was performed within 2 weeks of completion of an exercise intervention.

### Flow‐mediated dilatation

After 20 min supine rest, a 10 MHz linear‐array probe attached to a high‐resolution ultrasound machine (T3300, Terason, MA, USA) was used to assess femoral artery function in accordance with guidelines, using the flow‐mediated dilatation (FMD) technique (Thijssen et al., 2011). In short, a pneumatic cuff was wrapped around the thigh immediately above the knee. A 1 min baseline scan of the artery was obtained (by an experienced vascular sonographer) via continuous duplex ultrasound, to assess resting superficial femoral artery diameter and blood flow velocity. The blood flow‐occluding cuff was rapidly inflated to a suprasystolic pressure of 220 mmHg, which was sustained for 5 min. Image recording was resumed 30 s prior to cuff deflation and continued for 3 min post‐deflation. Analysis was performed as previously described (Thijssen et al., 2011; Woodman et al., 2001).

### Statistical analysis

Data presented in this paper are for 39 individuals who had both pre‐ and post‐intervention femoral vascular data for one or both modes of exercise training (mode ‘completers’). Statistical analyses were performed with SPSS v.29.0 (IBM Australia Ltd, NSW, Australia) and STATA v.17.0 software (StataCorp LLC, TX, USA). The effects of the exercise training interventions, and differences between the sexes for each outcome, were assessed using linear mixed models (LMMs), which adjust for the repeated nature of the data, controlling for individual fixed effects and age. LMMs account for the correlation between repeated measures from the same subject, allowing for more accurate estimates of treatment effects. Advantages of LMMs include the ability to handle non‐independence of observations attributable to repeated measures, unbalanced data and missing values (O'Connell et al., 2017). Our models have incorporated both fixed and random effects, which enhance the robustness of the models. In the present study, some data were missing due to participant withdrawal, illness, relocation or sonographer unavailability (see Fig. S1). A *z*‐test was used to assess the differences in individual response rates between exercise interventions.

## Results

Baseline characteristics of participants are included in Table 1. Attendance at training sessions was 94% for RES and 95% for END. There were no significant changes after RES or END training in daily caloric intake (*P* = 0.99 and *P* = 0.80, respectively), average energy expenditure (*P* = 0.60 and *P* = 0.22, respectively) or total energy expenditure (*P* = 0.42 and *P* = 0.24, respectively), as previously reported (Thomas et al., 2021).

The training was effective with regard to cardiorespiratory fitness and muscular strength results, also previously described (Marsh et al., 2020b; Thomas et al., 2021). Briefly, END, but not RES, significantly increased peak oxygen uptake (Δ3.61 ± 3.77  *vs*. 0.03 ± 3.57 ml kg^−1^ min^−1^, *P *< 0.001). Strength increased following RES but not END training (leg press, Δ47.0 ± 29.4 *vs*. 3.0 ± 26.4 kg, *P *< 0.001; bench press, Δ5.1 ± 5.0 *vs*. −0.4 ± 3.4 kg, *P *< 0.001). Males were stronger than females at entry to the study and, therefore, lifted more weight in absolute terms during the RES sessions, although the exercise prescriptions were personalized and matched in relative terms (i.e. %1RM). When 1RM data were divided by bodyweight, males were stronger per kilogram of bodyweight than females, and lifted more weight during RES sessions compared with females (see Table 1).

### Impact of RES and END on femoral vascular function: group means and individual responses

**Figure 1:**
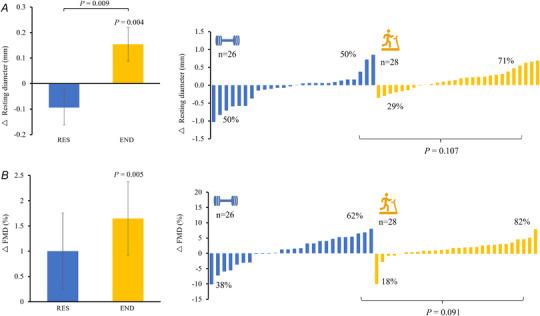
Changes in femoral resting artery diameter and flow‐mediated dilatation in response to 12 weeks of resistance and endurance exercise training Changes in femoral resting artery diameter (*A*) and flow‐mediated dilatation (*B*) in response to 12 weeks of resistance and endurance exercise training. Group mean responses are displayed in the left panel, with individual responses to RES (blue) and END (yellow) shown in the right panels. The *P*‐values on the waterfalls compare (*z*‐test) proportions of positive (>0) responders to RES *vs*. END, which are given in percentage terms on the plots. Abbreviations: Δ, change with exercise training; END, endurance; RES, resistance. [Colour figure can be viewed at wileyonlinelibrary.com]

**Figure 2:**
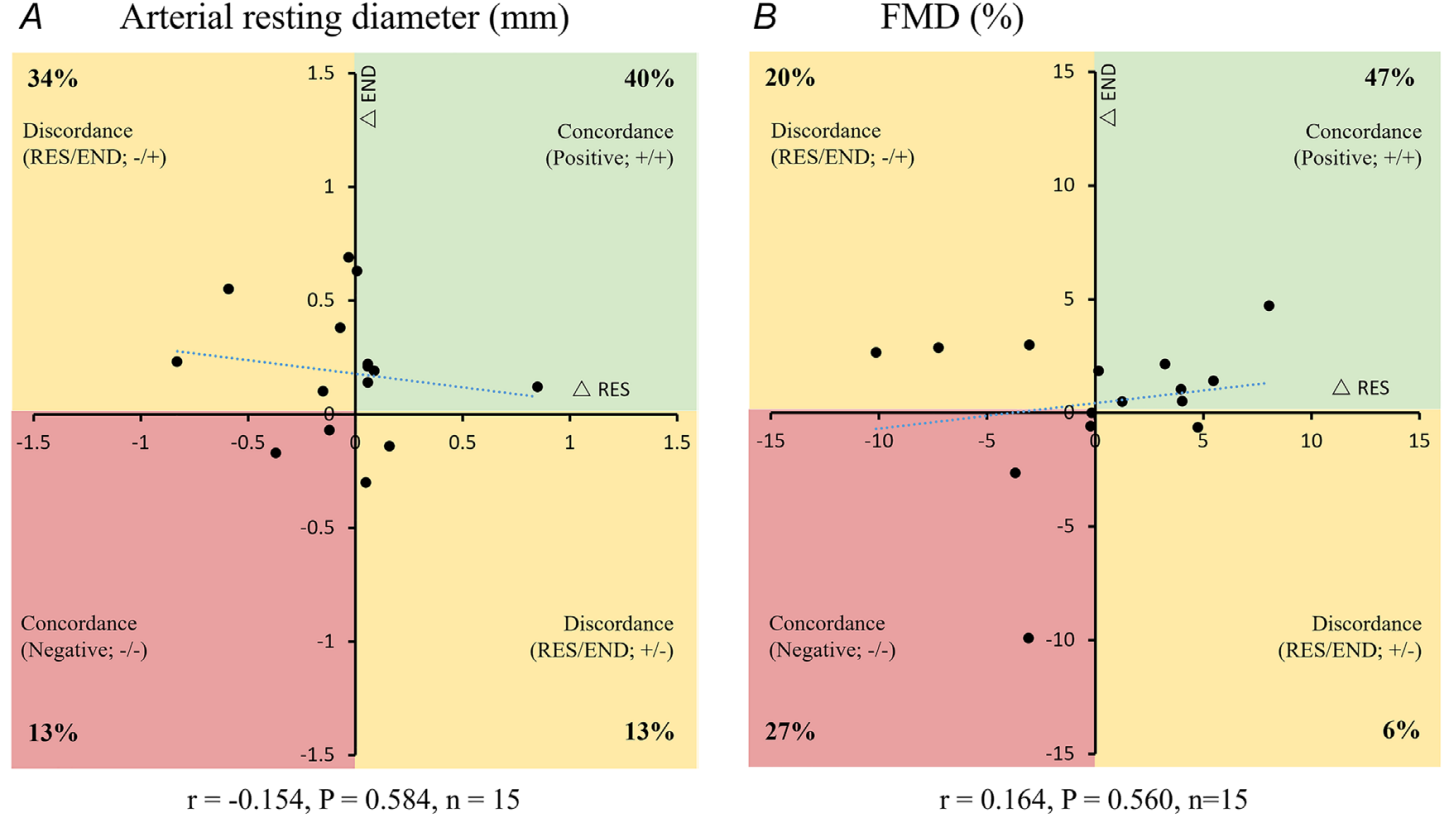
Individual participant exercise intervention change scores in femoral vascular parameters Individual participant exercise intervention change scores in femoral vascular parameters, where each dot represents the response by a given individual to both resistance (*x*‐axis) and endurance (*y*‐axis) training. *A*, change in femoral artery resting diameter. *B*, change in flow‐mediated dilatation. Percentages of responders are given in each quadrant. Abbreviations: Δ, change with exercise training; END, endurance; FMD, flow‐mediated dilatation; RES, resistance. [Colour figure can be viewed at wileyonlinelibrary.com]

Group mean responses to RES and END exercise training are presented in Table 2 and Fig. 1 (left panels), with all individual data shown in Fig. 1 (right panel waterfalls). Increases in both resting arterial diameter (Δ0.15 ± 0.29 mm, *P* = 0.004; Fig. 2*A*) and femoral FMD% (Δ1.61 ± 3.09%, *P = *0.005; Fig. 2*B*) were evident in response to END training, whereas no significant change occurred following RES (*P* > 0.260).

**Table 2:** Mean changes in femoral artery responses to resistance and endurance exercise training for males and females

Variable	Baseline		ΔExercise		*P*‐value
**Resistance (All)**	***n* = 26**		***n* = 26**		
Resting diameter (mm)	5.8 (1.0)		−0.09 (0.43)		
FMD (%)	6.6 (3.7)		0.90 (4.72)		
**Endurance (All)**	***n* = 28**		***n* = 28**		**RES *vs*. END (*P*‐value)**
Resting diameter (mm)	5.6 (1.0)		**0.15 (0.29)** ^a^		**0.009**
FMD (%)	7.2 (3.6)		**1.61 (3.09)** ^b^		0.466
**Resistance (by F *vs*. M)**	**F (*n* = 14)**	**M (*n* = 12)**	**F (*n* = 14)**	**M (*n* = 12)**	**F *vs*. M (*P*‐value)**
Resting diameter (mm)	5.11 (0.62)	6.55 (0.81)	−0.16 (0.42)	−0.02 (0.46)	0.407
FMD (%)	8.32 (3.67)	4.55 (2.71)	−0.21 (5.29)	**2.21 (3.76)** ^d^	0.140
**Endurance (by F *vs*. M)**	**F (*n* = 18)**	**M (*n* = 10)**	**F (*n* = 18)**	**M (*n* = 10)**	**F *vs*. M (*P*‐value)**
Resting diameter (mm)	5.04 (0.62)	6.58 (0.81)	0.11 (0.33)	**0.23 (0.20)** ^e^	0.307
FMD (%)	8.56 (3.58)	4.79 (2.27)	**1.88 (3.67)^c^**	**1.11 (1.65)** ^c^	0.520

Data are the mean (SD). Abbreviations: Δ, change with exercise training; END, endurance; F, female; FMD, flow‐mediated dilatation; M, male; RES, resistance.

^a^ *P = *0.004

^b^ *P = *0.005

^c^ *P = *0.025

^d^ *P* = 0.015

^e^ *P* < 0.001 (linear mixed models).

Analysis of individual response data showed positive FMD response rates (>0) to END and RES training of 82 and 62%, respectively (*P = *0.091 RES *vs*. END; Fig. 1*B* waterfalls). For resting arterial diameter, positive response rates were 71% (END) and 50% (RES; *P = *0.107 END *vs*. RES).

### Concordance of exercise response

The concordance plots displayed in Fig. 2 present paired responses for each individual to RES and END training. Data are therefore included for only those participants who had femoral FMD data for both RES and END modes of exercise training. For resting arterial diameter (Fig. 2*A*), 40% of individuals were positively concordant; that is, there was an increase in diameter in response to both RES training and END training (+/+; Fig. 2*A*, top right). Negative concordance was 13% (−/−; Fig. 2*A*, bottom left), and discordance (directionally different responses to the two exercise modes) was 13% (+/−) and 34% (−/+). Almost half (47%) of participants were positively concordant for FMD (Fig. 2*B*), with a negative concordance of 27% and discordant percentages of 6 and 20%.

There were no significant correlations between RES and END responses for either femoral FMD% (*r* = 0.164, *P = *0.560) or resting diameter (*r* = −0.154, *P = *0.584). Therefore, a high response to one mode of training did not necessarily imply a high response to the alternative mode, and a low response to one mode does not preclude a high response to the other.

### Sex differences in femoral vascular responses to RES and END

**Figure 3:**
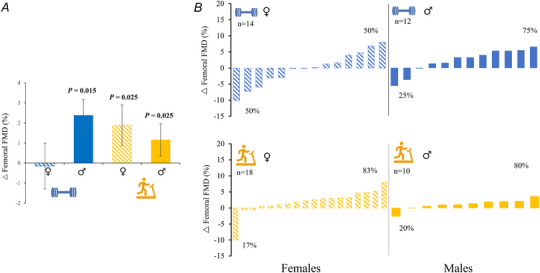
Sex differences in femoral vascular function with resistance and endurance training *A*, mean changes in flow‐mediated dilatation. *B*, individual data and pre–post changes. Abbreviations: Δ, change with exercise training; FMD, flow‐mediated dilatation. [Colour figure can be viewed at wileyonlinelibrary.com]

Sex‐specific group mean responses to RES and END training are presented in Table 2 and Fig. 3. In response to END training, males improved both resting diameter (0.23 ± 0.20 mm, *P* < 0.001) and FMD (1.11 ± 1.65%, *P* = 0.025). Males also increased FMD in response to RES training (2.21 ± 3.76%, *P* = 0.015). An increase in FMD following END training was also evident in females (1.88 ± 3.67%, *P* = 0.025); however, resting diameter did not change significantly (*P* = 0.124) in response to END. RES training did not elicit changes in either arterial resting diameter (−0.16 ± 0.42 mm, *P* = 0.148) or FMD (−0.21 ± 5.29%, *P* = 0.869) in females.

## Discussion

Vascular endothelial dysfunction is an early and integral atherosclerotic event (Moncada et al., 1991). Endothelial function can be enhanced by episodic increases in blood flow and shear stress, which trigger the upregulation of endothelium‐derived NO synthase, alongside other paracrine hormones, thereby enhancing endothelium‐dependent vasodilatation and decreasing atherosclerotic progression and risk (Green et al., 2017; Hambrecht et al., 2003; Pohl et al., 1986; Rubanyi et al., 1986). Exercise is an important physiological stimulus for preserving or upregulating endothelial function in humans (Green, O'Driscoll, Blanksby, et al., 1996; Hambrecht et al., 2000; Maiorana et al., 2001), and it has been proposed that exercise‐mediated improvement in endothelial function explains the observation that changes in traditional CV risk factors do not fully account for the beneficial impact of exercise on CV events (Green et al., 2008). In a series of ‘clamping’ experiments in humans, we established that endothelium‐ and NO‐dependent vascular functional and structural adaptation in response to interventions such as hand‐grip exercise, leg exercise, forearm heating and lower‐limb heating occur only when these interventions are accompanied by increases in intra‐arterial shear stress (Birk et al., 2012; Carter et al., 2014; Green et al., 2017; Naylor et al., 2011; Tinken et al., 2010). This suggests an obligatory role for shear stress in vascular adaptation, and other experiments indicate that the type (or pattern) of shear stress (anterograde *vs*. retrograde) modulates the adaptive response (Green et al., 2002, 2005, 2017). It follows that forms of exercise that induce distinct haemodynamic and shear stress patterns, such as RES *versus* END, might be associated with different adaptations.

Our principal finding is that END significantly increased baseline femoral artery diameter and FMD, whereas RES did not increase baseline femoral diameter (the difference between END and RES was significant) and did not significantly increase group responses to FMD. These findings suggest that END exercise has a larger impact on lower‐limb arterial adaptation than RES in humans. Given our previous findings relating to the impacts of shear stress, and the pattern of shear, on artery adaptation in humans (Green et al., 2017), it is tempting to speculate that END has more beneficial impacts because it is associated with prolonged elevation in anterograde shear in comparison to RES. It is relevant, in this context, that END involving cycling and running/walking persistently and systemically engages large muscle groups for the duration of the activity, and, in particular, lower‐limb muscle groups. In contrast, RES involves intermittent patterns of activity, with rest breaks, and in some cases, focuses on upper‐body muscle groups. Nonetheless, it should be noted that the 1% increase in FMD in response to RES can be considered clinically significant [based on brachial artery function studies (Green et al., 2011)], despite not achieving statistical significance in this study. Furthermore, a relatively large proportion of individuals responded positively to RES in terms of FMD change (62%, *vs*. 82% for END).

Visual inspection of individual responses to training (i.e. Fig. 1) reveals a wide range of individual responsiveness to both forms of training. This is generally consistent with recent human studies of exercise responsiveness (Bouchard et al., 2012; Green, Eijsvogels, Bouts, et al., 2014) to other physiological variables. Some evidence suggests that the characteristics of the exercise prescription (e.g. intensity) can modulate the ratio of responders to non‐responders (Montero & Lundby, 2017), and our study adds to these data, in that it indicates that differences exist in response rates in the same group of individuals when they are exposed to differing modalities of training.

The END‐induced increase in femoral artery baseline diameter might reflect adaptation that reduces peripheral resistance and contributes to blood pressure‐lowering effects of exercise, although it is not possible to attribute changes in femoral diameter directly to resistance artery remodelling or to distinguish structural adaptation from changes in basal arterial function (Naylor et al., 2005). It is also notable that femoral FMD increased substantially following END, despite an increase in baseline diameter, which would normally mitigate FMD‐related functional adaptation (Atkinson et al., 2009). Our findings are in keeping with previous evidence regarding the impacts of END training in humans (Ashor et al., 2015; Dawson et al., 2021; Green et al., 2017; Green, O'Driscoll, Blanksby, et al., 1996) and also with studies demonstrating that END athletes have enlarged arteries in comparison to matched control subjects, and that regional differences in arterial remodelling are apparent between active and inactive muscle beds within athletes (e.g. in racquet sports) (Green, Fowler, O'Driscoll, et al., 1996; Rowley et al., 2011, 2012).

**Figure 4:**
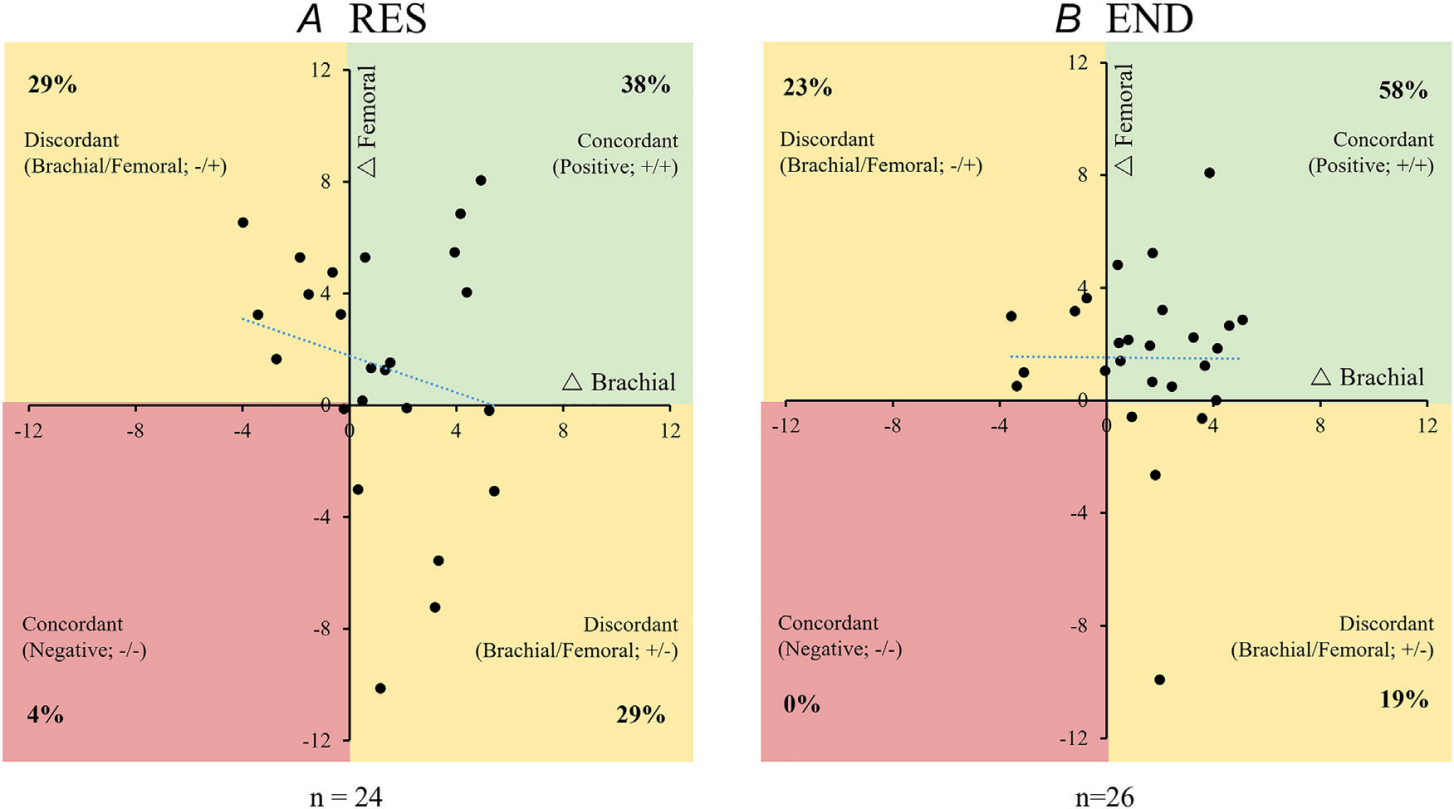
Individual participant exercise intervention change in brachial and femoral flow‐mediated dilatation Individual participant exercise intervention change in brachial and femoral flow‐mediated dilatation in response to resistance (*A*) and endurance (*B*) exercise training interventions. Each dot represents the FMD responses by a given individual in femoral (*y*‐axis) and brachial (*x*‐axis) flow‐mediated dilatation. Percentages of concordant *versus* discordant responses are provided in each quadrant. Abbreviations: Δ, change with exercise training; END, endurance; FMD, flow‐mediated dilatation; RES, resistance. [Colour figure can be viewed at wileyonlinelibrary.com]

Brachial artery FMD is largely mediated by NO (Green, Dawson, Groenewoud, et al., 2014), strongly correlated with coronary function and independently predicts cardiac events (Green et al., 2011). We have previously published outcomes from this experiment showing that both END and RES induce increases in brachial FMD (Green et al., 2023), consistent with other studies (Ashor et al., 2015; Dawson et al., 2021). In the present paper, both forms of exercise increased FMD by levels that have, on average, been associated with important clinical outcomes: a 1% change in brachial artery FMD is associated with ∼13% change in CV risk (Green et al., 2011). Nonetheless, the increase in FMD following RES (∼1%) was not significant in the femoral artery, in contrast to brachial artery measures (∼1.7%) (Green et al., 2023). This might reflect different intrinsic physiology between the upper‐ and lower‐limb conduit arteries or differential patterns of shear between the upper and lower limbs in response to RES, which involved some hand‐gripping and potential forearm reactive hyperaemia. It is notable that when we correlated matching brachial *vs*. femoral data in participants who had both sets of measures collected simultaneously (Fig. 4), FMD responses to neither RES nor END were correlated.

There were notable sex differences in response to femoral artery FMD in this study (Fig. 3). Males were more responsive to RES training than females, whereas both sexes responded positively, on average, to END. Males were more responsive to RES than END, whereas the opposite was true for females. These data differ somewhat from our brachial artery analyses of this study, with respect to the impact of RES in men (Green et al., 2023). Females exhibited larger responses than males in response to END in both arteries, and also to RES in the brachial artery, whereas males responded more to RES in the femoral artery. It might be relevant that males exhibit larger increases in skeletal muscle mass than females and that testosterone is associated with increases in FMD when combined with exercise (Chasland et al., 2021). Despite being matched in relative terms (i.e. %1RM), males in our cohort also lifted more weight in absolute terms during the RES sessions than females. In older individuals, Black et al. (2009) observed improvements in brachial FMD% in men but not women following 24 weeks of END, and other studies using briefer interventions and cross‐sectional comparisons endorsed this finding where women were post‐menopausal (Pierce et al., 2011). Moreau et al. (2013) have pointed out that FMD% responses to training might be oestradiol dependent, which is generally consistent with our study, which involved pre‐menopausal women, but our femoral artery findings suggest that younger non‐oestrogen‐deficient women benefit less from RES than END.

There were some differences between males and females at the start of the study in terms of FMD, which are consistent with sex differences observed in normative data sets (Holder et al., 2021). The relatively preserved FMD in females did not limit their capacity to improve FMD in response to END, although we cannot exclude the possibility that it might have done so to RES. In a previous paper involving individuals in this study, we comprehensively assessed changes in CV risk factors in response to each form of exercise training and compared these in males and females (Thomas et al., 2023). We found no evidence for differences between men and women in the changes observed in body composition, blood pressure, lipid profiles, blood glucose or insulin levels between males and females in response to either form of training. This makes it unlikely that changes in these factors explain the sex differences in FMD in the present study. Whatever the mechanisms, our observations of sex differences in response to commonly used and ecologically valid exercise interventions have implications for exercise prescription, particularly where vascular health is considered an important outcome.

This study has several limitations. We were unable to collect femoral and brachial data simultaneously in all volunteers, largely owing to the lack of availability of a second trained sonographer. Nonetheless, our group sample size is double that of most published studies on vascular adaptation to training, and our within‐subjects design further minimized sources of error that influence physiological comparisons between subjects. Our 12 week intervention is also longer than most previous studies, and the prolonged washout we used was designed to allow for variables to return to baseline levels between interventions (the first and second baseline values were not significantly different, *P* = 0.629). We have not presented glyceryl trinitrate data because, unlike the brachial artery, we did not consistently collect these data in the femoral artery and are underpowered for comparative analyses. Owing to the logistical and scheduling issues related to the exercise intervention and washout periods, it was not possible to match all female participants for the timing of the menstrual cycle. It is unlikely that the lack of an inactive control group compromises the findings of this superiority design, given the large number of previous studies that have included time controls and demonstrated no change in FMD. Finally, we cannot exclude the possibility that different exercise interventions to those we used might result in different outcomes. However, we adopted interventions, informed by international guidelines, that are ecologically valid in terms of the types of training typically used to target cardiopulmonary *vs*. skeletal muscle adaptation in community settings. The exercise load included in this study (i.e. 3 × 60 min sessions) is consistent with the WHO physical activity recommendations for adults (World Health Organization, 2020), which also incorporates strength and END modes of training.

In summary, our data revealed that END has greater impacts than RES training on femoral artery diameter and FMD responses, which are endothelium mediated and NO dependent. Males exhibited beneficial impacts in response to both END and RES, whereas females responded predominantly to END. Our findings suggest that arterial adaptation to exercise might be influenced by exercise modality and sex, with implications for exercise prescription, particularly where vascular health is considered an important outcome.

## Additional information

### Competing interests

None declared.

### Author contributions

All authors contributed to writing the paper or critically reviewed the manuscript. D.G. conceived and designed the experiment. H.T. and C.M. recruited participants. H.T., C.M., L.N. and A.H. collected and analysed data. L.L. performed statistical analysis. All authors approved the final version of the manuscript and agree to be accountable for all aspects of the work, in ensuring that questions related to the accuracy or integrity of any part of the work are appropriately investigated and resolved. All persons designated as authors qualify for authorship and all those who qualify for authorship are listed.

### Funding

Daniel J. Green was supported by an NHMRC Principal Research Fellowship (APP1080914) and a Western Australian FHRI REA award. Hannah J. Thomas was supported by an Exercise and Sport Science Australia (ESSA) Clinical Exercise Physiology Research Grant.

## Supporting information




Peer Review History

**Figure S1**. Consort diagram. Number of participants who had femoral scans available for each exercise mode. Abbreviations: END, endurance; F, female; M, male; RES, resistance.

## Data Availability

All data are included as individual data points in the figures contained in this manuscript.
