# Supplementary figures and images for: Impact of resistance and endurance exercise training on femoral artery function: sex differences in humans
Source: J Physiol. 2025 Feb 6;603(5):1045–56. doi: 10.1113/JP287534 (PMC11870055; doi:10.1113/JP287534)

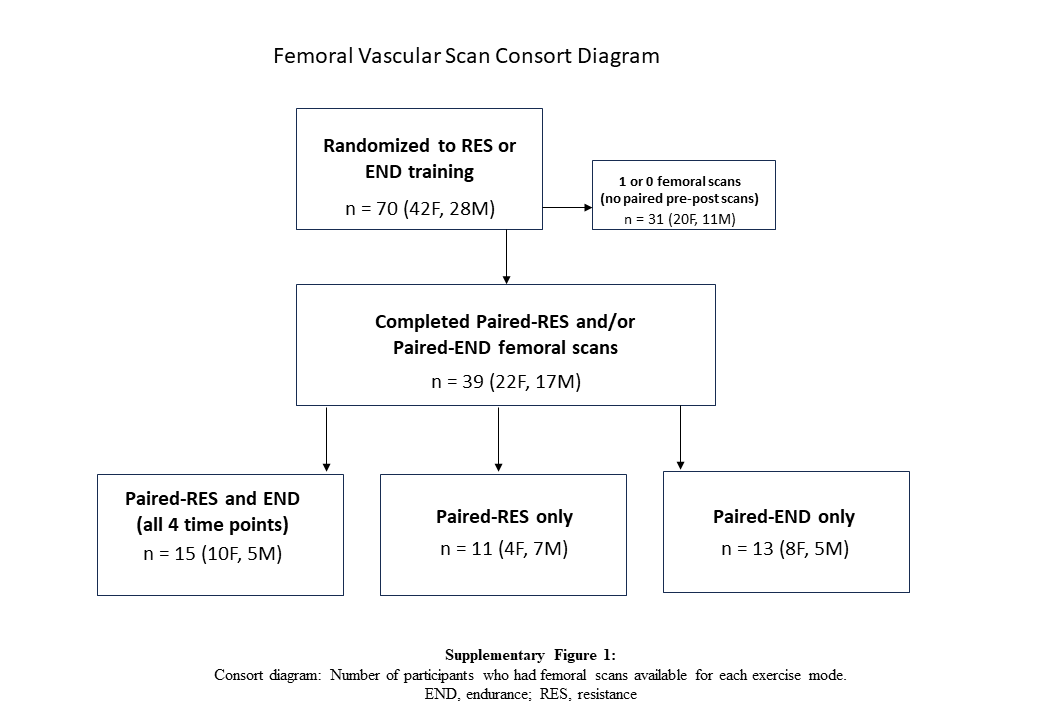

Supplement: Supplementary file 2 — Figure S1. Consort diagram. Number of participants who had femoral scans available for each exercise mode. Abbreviations: END, endurance; F, female; M, male; RES, resistance. [file TJP-603-1045-s001.tif]
