# Supplementary material for: Impact of resistance and endurance exercise training on femoral artery function: sex differences in humans
Source: J Physiol. 2025 Feb 6;603(5):1045–56. doi: 10.1113/JP287534 (PMC11870055; doi:10.1113/JP287534)
Supplement: Supplementary file 1 — Peer Review History [file TJP-603-1045-s002.pdf]

The Journal of Physiology

Impact of resistance and endurance exercise training on femoral artery function: sex differences in humans

Daniel J Green, Hannah J Thomas, Channa E Marsh, Leanne Lester, Louise Naylor, and Andrew Haynes
DOI: 10.1113/JP287534

Corresponding author(s): Daniel Green (danny.green@uwa.edu.au)

The referees have opted to remain anonymous.

Review Timeline:

Submission Date:	21-Aug-2024
Editorial Decision:	08-Oct-2024
Revision Received:	07-Nov-2024
Editorial Decision:	10-Jan-2025
Revision Received:	12-Jan-2025
Accepted:	15-Jan-2025

Senior Editor: Paul Greenhaff

Reviewing Editor: Bruno Grassi

Transaction Report:

(Note: With the exception of the correction of typographical or spelling errors that could be a source of ambiguity, letters and reports are not edited. Depending on transfer agreements, referee reports obtained elsewhere may or may not be included in this compilation. Referee reports are anonymous unless the Referee chooses to sign their reports.)

Dear Dr Green,

Re: JP-RP-2024-287534 "Impact of resistance and endurance exercise training on femoral artery function: sex differences in humans" by Daniel J. Green, Hannah J Thomas, Channa E Marsh, Leanne Lester, Louise Naylor, and Andrew Haynes

Thank you for submitting your manuscript to The Journal of Physiology. It has been assessed by a Reviewing Editor and by 2 expert referees and we are pleased to tell you that it is potentially acceptable for publication following satisfactory major revision.

Please advise your co-authors of this decision as soon as possible.

The referee reports are copied at the end of this email.

Please address all the points raised and incorporate all requested revisions or explain in your Response to Referees why a change has not been made. We hope you will find the comments helpful and that you will be able to return your revised manuscript within 9 months. If you require longer than this, please contact journal staff: jp@physoc.org. Please note that this letter does not constitute a guarantee for acceptance of your revised manuscript.

Your revised manuscript should be submitted online using the link in your Author Tasks: Link Not Available. This link is accessible via your account as Corresponding Author; it is not available to your co-authors. If this presents a problem, please contact journal staff (jp@physoc.org). Image files from the previous version are retained on the system. Please ensure you replace or remove any files that are being revised.

If you do not wish to submit a revised version of your manuscript, you must inform our journal staff (jp@physoc.org) or reply to this email to request withdrawal. Please note that a manuscript must be formally withdrawn from the peer review process at one journal before it may be submitted to another journal.

TRANSPARENT PEER REVIEW POLICY: To improve the transparency of its peer review process, The Journal of Physiology publishes online as supporting information the peer review history of all articles accepted for publication. Readers will have access to decision letters, including Editors' comments and referee reports, for each version of the manuscript, as well as any author responses to peer review comments. Referees can decide whether or not they wish to be named on the peer review history document.

ABSTRACT FIGURES: Authors are expected to use The Journal's premium BioRender account to create/redraw their Abstract Figures. Information on how to access this account is here: https://physoc.onlinelibrary.wiley.com/journal/14697793/biorender-access.

This will enable Authors to create and download high-resolution figures. If authors have used the free BioRender service, they can use the instructions provided in the link above to download a high-resolution version suitable for publication.

The link provided should only be used for the purposes of this submission. Authors will be charged for figures created on this account if they are not related to this manuscript submission.

LANGUAGE EDITING AND SUPPORT FOR PUBLICATION: If you would like help with English language editing, or other article preparation support, Wiley Editing Services offers expert help, including English Language Editing, as well as translation, manuscript formatting, and figure formatting at www.wileyauthors.com/eeo/preparation. You can also find resources for Preparing Your Article for general guidance about writing and preparing your manuscript at www.wileyauthors.com/eeo/prepresources.

RIGOUR AND REPRODUCIBILITY: We recommend authors consult The Journal's Rigour and Reproducibility requirements to ensure that their manuscript is suitable for publication: https://physoc.onlinelibrary.wiley.com/pb-assets/hub-assets/physoc/documents/TJP-Rigour-and-Reproducibility-Requirements.pdf

REVISION CHECKLIST:

Check that your Methods section conforms to journal policy: https://jp.msubmit.net/cgi-bin/main.plex?form_type=display_requirements#methods.

Check that data presented conforms to the statistics policy: https://jp.msubmit.net/cgi-bin/main.plex?form_type=display_requirements#statistics.

Upload a full Response to Referees file. To create your 'Response to Referees': copy all the reports, including any comments from the Senior and Reviewing Editors, into a Microsoft Word, or similar, file and respond to each point, using font or background colour to distinguish comments and responses and upload as the required file type.

Please upload two versions of your manuscript text: one with all relevant changes highlighted and one clean version with no

changes tracked. The manuscript file should include all tables and figure legends, but each figure/graph should be uploaded as separate, high-resolution files.

You may also upload:

- 'Potential Cover Art' for consideration as the issue's cover image
- Appropriate Supporting Information (Video, audio or data set: see https://jp.msubmit.net/cgi-bin/main.plex?form_type=display_requirements#supp).

We look forward to receiving your revised submission.

If you have any queries, please reply to this email and we will be pleased to advise.

Yours sincerely,

Paul Greenhaff
Senior Editor
The Journal of Physiology

EDITOR COMMENTS

Reviewing Editor:

Comments to the Author:
The study evaluates the impact of resistance ad endurance training in humans of femoral artery function, with specific attention to sex differences. The study addresses an important physiological question and the findings are of interest. Both Reviewers raise some criticisms which should be convincingly addressed by the authors.

Reviewer 1 main concerns point to reference to previous studies, to the number of drop-out subjects, and to other issues which should be better addressed in the Discussion. Reviewer 2 points to specific methodological and data interpretation issues, as nicely outlined in her/his response.

Senior Editor:

Comments for Authors to ensure the paper complies with the Statistics Policy (Required):
Table 1 F vs M P-values need to be = not <

If the research study was registered (clause 35 of the Declaration of Helsinki) the registration database should be indicated, else the lack of registration should be noted as an exception (e.g. The study conformed to the standards set by the Declaration of Helsinki, except for registration in a database.)

Thank you for the manuscript submission to The Journal of Physiology, which has been considered by a reviewing editor and two reviewers. All believe the study addresses a topic that is interesting. However, there are a number of major concerns that the authors need to consider, including the inclusion of additional data. At the moment Reviewer 2 is not convinced about the scientific impact of the study and therefore the authors need to address all points from both reviewers comprehensively when revising the manuscript.

REFEREE COMMENTS

Referee #1:

The study by Green et al asks an important physiological question, and the findings offer important knowledge to the exercise physiology literature. The manuscript is scientifically sound and is part of a series of results from a larger suite of data. The data are original and the conclusions are based on appropriate data modelling. I have some comments and suggestions for the authors to consider which are outlined below.

The manuscript references and refers in places to other publications for information including Marsh et al., Thomas et al., and Green et al. These previously published manuscripts are from a twin study (STRUTH), it is not clear in the current manuscript how this data is linked to this study. Are the current data set twin pairs? More clarity needed throughout (e.g. lines 9, 62, 75, 76). In addition, whilst it is useful to refer to other previously published studies to not repeat detailed methods and results, there are instances where more information is required to help the reader. Lines 87 - was PA and diet measured/recorded? Lines 94-100- more detail on the type of resistance exercise required including exercises, body parts and how performed (barbell/resistance machine etc). This is specifically important in this manuscript, how much leg exercise was performed (reps, sets, weights), given the primary outcome of femoral FMD/diameter.

The manuscript starts in the abstract and methods with 39 participants but in the results and figures the numbers are lower e.g. n=26 and 28 in figure 1. It is unclear reasons for drop out or how recruitment of these individuals happened. A consort type schematic figure would assist the reader. Moreover in figures 2 and 4 the n would be useful to include on figure or in the legend.

The reference in the discussion that a 1% change in FMD could be clinically significant needs some explanation that this data is specific to brachial artery. Clinical significance may be different for femoral (maybe even lower that 1%) in femoral given the regional differences (e.g. size).

Consider in the discussion, some comparison of the differences in actual exercise time between the END and RES exercise sessions. For example, END of cycling and running/walking is whole body exercise that requires movement/force for the duration of the exercise and is by nature a largely lower limb exercise. RES would not require the same movement/force time and the prescription is not limited to lower body RES workouts.

Also consider in the discussion of the comparison of males and females, the concept that males may have been able to lift more load relative to their body weight than females despite calculations and consideration %RM. Or in other words is it conceivable that males lifted more weight relative to body weight for leg exercise? It might be possible to calculate this from your data.

If the study has implications for exercise prescription as outlined in the key points on page 3, then the above two points need some consideration. Moreover in this section more insight into potential implications for exercise prescription would be helpful i.e. are you inferring that females should not do RES exercise to improve vascular health? Can you make specific recommendations about exercise prescription from this data?

Lines 56 -refers to previous studies but no references included.

Lines 78-80 outlines that brachial and femoral FMDs performed at same time (with no time delay offset). Any data/insight as to if cuff inflation of arm and leg simultaneously has an impact haemodynamically?

Lines 102-105. Where females tested at specific part of menstrual cycle? Information should be included in manuscript.

Lines 123-126. It would help the reader to outline how the LMM were built for analysis in more detail.

Referee #2:

In the present work authors examine the effects of 12 weeks of endurance and resistance training on baseline arterial diameter and flow-mediated vasodilation in 39 young healthy participants. Authors demonstrated endurance training had a bigger impact as compared with resistance training. Furthermore, sex-difference in response was noted with males and females responding to endurance training and males only to resistance training.

This paper is interesting. However, there are couple of issues that should be address before this paper is published.

1/ Male participants had a weaker FMD response at the starting point, as compared with females (4.55 vs 8.32 , and 4.79 vs 8.56 for two types of interventions, respectively), and had higher BMI (26. Vs 21.8). Could this be a reason for the difference? It would be advisable to provide some additional results to confirm that the male participant do not have risks factors for CVD that could explain lower FMD response as compared with females.

2/ Figure 1 nicely shows individuals responses of arterial baseline diameter and FMD to training. What is the reason for such a high individual variation in the response to exercise? IT would be interesting to add some thoughts of the authors to the discussion.

3/ The exercise protocol of endurance training was 3x1 hour sessions per week for 12 weeks. How this related to WHO recommendation and health effects of exercise described by others More elaborated comments on this in the discussion would be advisable to know the opinion of the authors on the extent of endurance exercise that is optimal for the improvement of endothelial function in comparison with other studies.

4/ Authors did not measure FMD in the arm, only in the leg, and consider this as a major limitation of the study. Some comparison of their results with effects on FMD in brachial artery are given in the discussion. These aspects should be better discussed. The review is not certain, whether the effect described can be considered systemic or is local?

5/Authors did not include basal clinical biochemistry data of the participants. Some additional results would strength the paper.

(Impact of resistance and endurance exercise training on femoral artery function: sex differences in humans

Daniel J. Green[1], Hannah J. Thomas[1], Channa E. Marsh[1], Leanne Lester[1,2], Louise H. Naylor[1], Andrew Haynes[1]

[1]School of Human Sciences (Exercise and Sport Science),
and [2]Business School (Centre for Social Impact),
The University of Western Australia, Perth, Western Australia, Australia

Running title: Exercise modalities and femoral artery function

TOC Category: Cardiovascular

Author for correspondence:

Winthrop Professor Daniel J Green

School of Human Sciences (Exercise and Sport Science),

The University of Western Australia, Perth, WA, Australia, 6009

danny.green@uwa.edu.au, Phone +61 8 6488 2361

ORCID 0000-0003-3226-2921

We thank the Editors and reviewers for their helpful insight, feedback and suggestions. We have endeavoured to incorporate this feedback into the manuscript. Our responses to reviewer comments and changes made to the manuscript are detailed in the table below.

Editor Comments:

Comments: The study evaluates the impact of resistance and endurance training in humans of femoral artery function, with specific attention to sex differences. The study addresses an important physiological question and the findings are of interest. Both Reviewers raise some criticisms which should be convincingly addressed by the authors.

Reviewer 1 main concerns point to reference to previous studies, to the number of drop-out subjects, and to other issues which should be better addressed in the Discussion.

Reviewer 2 points to specific methodological and data interpretation issues, as nicely outlined in her/his response

Thank you for the manuscript submission to The Journal of Physiology, which has been considered by a reviewing editor and two reviewers. All believe the study addresses a topic that is interesting. However, there are a number of major concerns that the authors need to consider, including the inclusion of additional data. At the moment Reviewer 2 is not convinced about the scientific impact of the study and therefore the authors need to address all points from both reviewers comprehensively when revising the manuscript.

Response: Thank you for the opportunity to revise and resubmit this work. We think we have comprehensively addressed all concerns.

Editor	Comment	Response
	Comments for Authors to ensure the paper complies with the Statistics Policy (Required):<br>Table 1 F vs M P-values need to be = not <	**Thank you. The Table and text now contain "P= " to 3 decimal places. Beyond this level, we used p<0.001. This is consistent with journal's statistics policy.**
	If the research study was registered (clause 35 of the Declaration of Helsinki) the registration database should be indicated, else the lack of registration should be noted as an exception (e.g. The study conformed to the standards set by the Declaration of Helsinki, except for registration in a database.)	**Thank you for bringing this point to our attention. The registration database has now been added to the second sentence in the Methods.**<br>*Study registration with the Australian New Zealand Clinical Trials Registry (ACTRN12616001095459) was completed prior to randomisation.*

Reviewer #1 Summary Comments:
The study by Green et al asks an important physiological question, and the findings offer important knowledge to the exercise physiology literature. The manuscript is scientifically sound and is part of a series of results from a larger suite of data. The data are original, and the conclusions are based on appropriate data modelling. I have some comments and suggestions for the authors to consider which are outlined below.
We are very grateful for your knowledgeable, helpful, and constructive review. We have made every effort to make the changes you requested.

R1	Comment	Response
#1	The manuscript references and refers in places to other publications for information including Marsh et al., Thomas et al., and Green et al. These previously published manuscripts are from a twin study (STRUTH), it is not clear in the current manuscript how this data is linked to this study. Are the current data set twin pairs?	**The data in this manuscript are indeed derived from our STRUETH study of twin pairs. Femoral FMD was only collected in a sub-sample of those participants.**<br>**We have added information in the manuscript to clarify this point.**<br>**Methods under sub-heading Participants:**<br>*Data presented in this paper relate to a cross-over exercise intervention study of twin pairs, involving 70 healthy but relatively inactive young adult participants (i.e. 35 twin-pairs). Participants were aged 26.9 ± 6.2 years, non-smokers, self-reported no cardiovascular or other diseases, and did not meet the Australian guidelines for physical activity of 150-mins per week. Due to the lower number of recruited pairs who completed the 3 femoral artery scans (due to unavailability of a second sonographer), heritability analyses have not been included in this manuscript.*

**#2**	In addition, whilst it is useful to refer to other previously published studies to not repeat detailed methods and results, there are instances where more information is required to help the reader. Lines 87 - was PA and diet measured/recorded?	**PA and diet diaries were collected, and data were reported in Thomas et al. MSSE 53: 58-67, 2021. We have added reference to the methods we used paper and also referred to the results previously reported in that paper:**<br>*Physical activity was monitored (Actiheart 4; CamNtech Ltd., Cambridge, UK) to assess total (TEE) and active (AEE) energy expenditure in the week before or immediately after each exercise intervention to quantify incidental changes in physical activity levels. Participants wore the monitor for a 5-d period. A food diary was also given to each participant to complete the week before, or immediately after, each exercise intervention. Participants were instructed to complete the diary on four consecutive days, including one weekend day. Mean total daily energy intake was determined from these records using a commercially available software program (Foodworks 9; Xyris Software, Queensland, Australia).*<br><br>*There were no significant changes after RES or END training in daily caloric intake (P = 0.99 and P = 0.80, respectively), average energy expenditure (P = 0.60 and P = 0.22, respectively), or total energy expenditure (P = 0.42 and P = 0.24, respectively), as previously reported (Thomas et al., 2021).*

**#3**	Lines 94-100- more detail on the type of resistance exercise required including exercises, body parts and how performed (barbell/resistance machine etc). This is specifically important in this manuscript, how much leg exercise was performed (reps, sets, weights), given the primary outcome of femoral FMD/diameter.	**Thank you. Exercises were primarily compound exercises using free weights, but also included a combination of machines. We have now added more information to the Methods under the heading "Exercise Interventions" related to the exercise protocols.**<br>*RES alternated each session between upper body (i.e. bench press, standing military press) and lower body (i.e. barbell back squats, deadlifts, leg press), progressing from 60-90% of one-repetition maximum with repetitions decreasing from 15 – 5 reps as load increased . For interpretation of strength and exercise load data, 1RM results are also presented relative to body weight.*

**#4**	The manuscript starts in the abstract and methods with 39 participants but in the results and figures the numbers are lower e.g. n=26 and 28 in figure 1. It is unclear reasons for drop out or how recruitment of these individuals happened. A consort type schematic figure would assist the reader. Moreover, in figures 2 and 4 the n would be useful to include on figure or in the legend.	**We have added a consort figure as an addendum to this manuscript. The main reason for missing data was that we had difficulty securing the services of a second trained sonographer to complete some of the scans (brachial and femoral scans were collected simultaneously and where only one scanner was available, we prioritised brachial scanning). We have also added the n values on the figures.**<br>**One reason that n values differ is that we performed statistical analyses in some cases on paired data before and after one or other of the interventions, whereas in other cases (e.g. figures 2 and 4) data are included where only those individuals with femoral data at all 4 timepoints of training are included (or femoral *and* brachial measures were available). This is all clarified in the Consort diagram you requested.**<br>**Added to the stats section:**<br>*The effects of the exercise training interventions, and differences between the sexes for each outcome, were assessed using linear mixed models (LMM) which adjusts for the repeated nature of the data, controlling for individual fixed effects and age. LMM accounts for correlation between repeated measures from the same subject, allowing for more accurate estimates of treatment effects. Advantages of LMM models include the ability to handle non-independence of observations due to repeated measures, unbalanced data and missing values (O'Connell et al., 2017).. Our models have incorporated both fixed and random effects, which enhance the model's robustness. In the current study, some data were missing due to participant withdrawal, illness, relocation, or sonographer unavailability (see Supplemental Consort diagram).*

**#5**	Consider in the discussion, some comparison of the differences in actual exercise time between the END and RES exercise sessions. For example, END of cycling and running/walking is whole body exercise that requires movement/force for the duration of the exercise and is by nature a largely lower limb exercise. RES would not require the same movement/force time and the prescription is not limited to lower body RES workouts.	**We have now addressed your point regarding the contrasts between END and RES exercise modalities in the Discussion.** *It is also relevant that END involving cycling and running/walking engages the large muscle groups systemically for the duration of the exercise, in particular lower limb muscle groups. In contrast, RES involves intermittent patterns of activity, with rest breaks, is not limited to lower body muscle groups.*
**#6**	Also consider in the discussion of the comparison of males and females, the concept that males may have been able to lift more load relative to their body weight than females despite calculations and consideration %RM. Or in other words is it conceivable that males lifted more weight relative to body weight for leg exercise? It might be possible to calculate this from your data.	**The exercises during strength training sessions were set at the same % of 1RM for all participants, in keeping with standard exercise physiology research practice.**<br>**But we have now added baseline strength test results to Table 1, which indicate that males were indeed stronger than females and also lifted more absolute weight (kg). We have added this information to the Methods, Results and Discussion.**<br>**Methods under sub-heading "Exercise Interventions":** *For interpretation of strength and exercise load data, 1RM results are also presented relative to body weight.*<br>**Results:** *Males were stronger than females at entry to the study and therefore lifted more weight in absolute terms during the RES sessions, even though the exercise prescriptions were personalised and matched in relative terms (i.e. %1RM). When 1RM data was divided by bodyweight, males were stronger per kg of bodyweight than females and lifted more relative weight during RES sessions compared to females (see Table 1).*<br>**Discussion:** *Despite being matched in relative terms (i.e. %1RM), males in our cohort also lifted more weight in absolute terms during the RES sessions than females.*

**#7**	If the study has implications for exercise prescription as outlined in the key points on page 3, then the above two points need some consideration. Moreover, in this section more insight into potential implications for exercise prescription would be helpful i.e. are you inferring that females should not do RES exercise to improve vascular health? Can you make specific recommendations about exercise prescription from this data?	**We have added a statement to the end of this paragraph to emphasise your point about the relevance of sex differences, which requires further study.** *our observations of sex differences in the response to commonly utilised and ecologically valid exercise interventions have implications for exercise prescription, particularly where vascular health is considered an important outcome.*
**#8**	Lines 56 -refers to previous studies but no references included	**Thank you, we have deleted the reference to previous studies.**
**#9**	Lines 78-80 outlines that brachial and femoral FMDs performed at same time (with no time delay offset). Any data/insight as to if cuff inflation of arm and leg simultaneously has an impact haemodynamically?	**We are unaware of any studies that have investigated the impact of simultaneous FMD measurement in the brachial and femoral arteries. If there is an impact on haemodynamics (e.g. BP, HR), then it would logically be the same in both arteries given that we assess the measures simultaneously. We have added a statement to the methods:** *We consider that performing upper and lower limb vascular measures simultaneously is a valid experimental approach, as it controls for any time-related differences in subject status and presentation.*
**#10**	Lines 102-105. Were females tested at specific part of menstrual cycle? Information should be included in manuscript.	**For logistical reasons, we did not control for testing according to timing of the menstrual cycle. We have added a statement to the limitations paragraph of the Discussion to make this clear.** *Due to the logistical and scheduling issues related to the exercise intervention and washout periods, it was not possible to match all female participants for timing of menstrual cycle.*
**#11**	Lines 123-126. It would help the reader to outline how the LMM were built for analysis in more detail.	**Thank you. We have added details to the stats section regarding LMM procedures.**

Reviewer #2 Summary Comments:
In the present work authors examine the effects of 12 weeks of endurance and resistance training on baseline arterial diameter and flow-mediated vasodilation in 39 young healthy participants. Authors demonstrated endurance training had a bigger impact as compared with resistance training. Furthermore, sex-difference in response was noted with males and females responding to endurance training and males only to resistance training.

This paper is interesting. However, there are couple of issues that should be address before this paper is published.
Thank you for your very detailed and incisive comments. They have undoubtedly improved our manuscript.

R2	Comment	Response
**#1**	Male participants had a weaker FMD response at the starting point, as compared with females (4.55 vs 8.32 , and 4.79 vs 8.56 for two types of interventions, respectively), and had higher BMI (26. Vs 21.8). Could this be a reason for the difference? It would be advisable to provide some additional results to confirm that the male participant do not have risks factors for CVD that could explain lower FMD response as compared with females.	**We have added a statement to the discussion regarding the possibility that differences in risk factors impacted the changes in sex differences in FMD that we observed.** *There were some differences between males and females at the start of the study in terms of FMD, which are consistent with sex differences observed in normative data sets. The relatively preserved FMD in females did not limit their capacity to improve FMD in response to END, although we cannot exclude the possibility that it may have done so to RES. In a previous study we comprehensively assessed changes in CV risk factors to each form of exercise training and compared these in males and females. We found no evidence for differences between men and women in the changes observed in body composition, blood pressure, lipid profiles, blood glucose or insulin levels between males and females to either form of training. This makes it unlikely that changes in these factors explain the sex differences in FMD in the present study.*

**#2**	Figure 1 nicely shows individuals responses of arterial baseline diameter and FMD to training. What is the reason for such a high individual variation in the response to exercise? It would be interesting to add some thoughts of the authors to the discussion.	**In recent years it has become increasingly clear that a large range of response occurs to exercise interventions. We have added a sentence to the Discussion to highlight this.**<br>*Visual inspection of individual responses to training (i.e. Figure 1) supports wide a range of individual responsiveness to both forms of training. This is generally consistent with recent human studies of exercise responsiveness to other physiological variables. Some evidence suggests that the characteristics of the exercise prescription, (e.g. intensity) can modulate the number of responders:non-responders, and our study adds to this data in that it indicates that differences exist in response rates in the same group of individuals when they are exposed to differing modalities of training.*
**#3**	The exercise protocol of endurance training was 3x1 hour sessions per week for 12 weeks. How this related to WHO recommendation and health effects of exercise described by others More elaborated comments on this in the discussion would be advisable to know the opinion of the authors on the extent of endurance exercise that is optimal for the improvement of endothelial function in comparison with other studies.	*We have now added information to the Discussion comparing our exercise interventions to current PA guidelines and the reasons we chose our interventions.*<br>*Finally, we cannot exclude the possibility that different exercise interventions to those we utilised might result in different outcomes. However, we adopted interventions informed by international guidelines, and that are ecologically valid in terms of the forms of training typically used to target cardiopulmonary versus skeletal muscle adaptation in community settings. The exercise load included in this study (i.e. 3 x 60 min sessions) is consistent with the WHO physical activity recommendations for adults (World Health Organization, 2020), which also incorporates strength and endurance modes of training.*
**#4**	Authors did not measure FMD in the arm, only in the leg, and consider this as a major limitation of the study. Some comparison of their results with effects on FMD in brachial artery are given in the discussion. These aspects should be better discussed. The review is not certain, whether the effect described can be considered systemic or is local?	**We assessed FMD in the brachial artery and have included these data where they are relevant to comparison with our femoral measures, the focus of the current manuscript (e.g. Figure 4 and in the Discussion). We did not emphasise the brachial data, *per se*, because they are included in a previous publication (which we have referred to).**

**#5**	Authors did not include basal clinical biochemistry data of the participants. Some additional results would strength the paper.	**We have added a statement to the discussion referring to a previous paper that contained comprehensive risk factor profiles in these from the participants in this study.** *In a previous study we comprehensively assessed changes in CV risk factors to each form of exercise training and compared these in males and females. We found no evidence for differences between men and women in the changes observed in body composition, blood pressure, lipid profiles, blood glucose or insulin levels between males and females to either form of training. This makes it unlikely that changes in these factors explain the sex differences in FMD in the present study. Whatever the mechanisms, our observations of sex differences in the response to commonly utilised and ecologically valid exercise interventions have implications for exercise prescription, particularly where vascular health is considered an important outcome.*

Dear Dr Green,

Re: JP-RP-2024-287534R1 "Impact of resistance and endurance exercise training on femoral artery function: sex differences in humans" by Daniel J Green, Hannah J Thomas, Channa E Marsh, Leanne Lester, Louise Naylor, and Andrew Haynes

Thank you for submitting your manuscript to The Journal of Physiology. It has been assessed by a Reviewing Editor and by 3 expert referees and we are pleased to tell you that it is acceptable for publication following satisfactory revision.

The referee reports are copied at the end of this email.

Please address all the points raised and incorporate all requested revisions or explain in your Response to Referees why a change has not been made. We hope you will find the comments helpful and that you will be able to return your revised manuscript within 4 weeks. If you require longer than this, please contact journal staff: jp@physoc.org.

Your revised manuscript should be submitted online using the link in your Author Tasks Link Not Available. This link is accessible via your account as Corresponding Author; it is not available to your co-authors. If this presents a problem, please contact journal staff (jp@physoc.org). Image files from the previous version are retained on the system. Please ensure you replace or remove any files that are being revised.

If you do not wish to submit a revised version of your manuscript, you must inform our journal staff (jp@physoc.org) or reply to this email to request withdrawal. Please note that a manuscript must be formally withdrawn from the peer review process at one journal before it may be submitted to another journal.

TRANSPARENT PEER REVIEW POLICY: To improve the transparency of its peer review process The Journal of Physiology publishes online, as supporting information, the peer review history of all articles accepted for publication. Readers will have access to decision letters, including Editors' comments and referee reports, for each version of the manuscript, as well as any author responses to peer review comments. Referees can decide whether or not they wish to be named on the peer review history document.

ABSTRACT FIGURES: Authors are expected to use The Journal's premium BioRender account to create/redraw their Abstract Figures. Information on how to access this account is here: https://physoc.onlinelibrary.wiley.com/journal/14697793/biorender-access.

This will enable Authors to create and download high-resolution figures. If authors have used the free BioRender service, they can use the instructions provided in the link above to download a high-resolution version suitable for publication.
The link provided should only be used for the purposes of this submission. Authors will be charged for figures created on this account if they are not related to this manuscript submission.

LANGUAGE EDITING AND SUPPORT FOR PUBLICATION: If you would like help with English language editing, or other article preparation support, Wiley Editing Services offers expert help, including English Language Editing, as well as translation, manuscript formatting, and figure formatting at www.wileyauthors.com/eeo/preparation. You can also find resources for Preparing Your Article for general guidance about writing and preparing your manuscript at www.wileyauthors.com/eeo/prepresources.

RIGOUR AND REPRODUCIBILITY: We recommend authors consult The Journal's Rigour and Reproducibility requirements to ensure that their manuscript is suitable for publication: https://physoc.onlinelibrary.wiley.com/pb-assets/hub-assets/physoc/documents/TJP-Rigour-and-Reproducibility-Requirements.pdf

REVISION CHECKLIST:
Check that your Methods section conforms to journal policy: https://jp.msubmit.net/cgi-bin/main.plex?form_type=display_requirements#methods

Check that data presented conforms to the statistics policy: https://jp.msubmit.net/cgi-bin/main.plex?form_type=display_requirements#statistics

Upload a full Response to Referees file. To create your 'Response to Referees' copy all the reports, including any comments from the Senior and Reviewing Editors, into a Microsoft Word, or similar, file and respond to each point, using font or background colour to distinguish comments and responses and upload as the required file type.

Please upload two versions of your manuscript text: one with all relevant changes highlighted and one clean version with no changes tracked. The manuscript file should include all tables and figure legends, but each figure/graph should be uploaded as separate, high-resolution files. The journal is now integrated with Wiley's Image Checking service. For further details, see: https://www.wiley.com/en-us/network/publishing/research-publishing/trending-stories/upholding-image-integrity-wileys-image-screening-service

You may also upload:
- 'Potential Cover Art' for consideration as the issue's cover image
- Appropriate Supporting Information (Video, audio or data set: see https://jp.msubmit.net/cgi-bin/main.plex?form_type=display_requirements#supp).

We look forward to receiving your revised submission.

If you have any queries, please reply to this email and we will be pleased to advise.

Yours sincerely,

Paul Greenhaff
Senior Editor
The Journal of Physiology

EDITOR COMMENTS

Reviewing Editor:

Comments to the Author:
The original two Reviewers were split: Reviewer 1 was satisfied with the revision and suggested acceptance, whereas Reviewer 2 was not convinced by the responses and suggested rejection. An experienced third reviewer was therefore invited as a tie-breaker. The third Reviewer judged "satisfactory" the revisions and the answers forwarded by the authors.

Senior Editor:

Comments to the Author:
Thank you for the revised manuscript which has been considered by the same reviewers that considered the original submission, and also a third reviewer due to a difference of opinion between Reviewer 1 and 2 regarding the revisions. I am happy to say that the majority opinion is that the paper should be accepted for publication in The Journal of Physiology. However, there is one further point that needs to be addressed please. The authors state in their response to the reviewers that "the data in this manuscript are indeed derived from our STRUETH study of twin pairs. Femoral FMD was only collected in a sub-sample of those participants. We have added information in the manuscript to clarify this point", but it appears that this information is not overtly clear in the manuscript. Please therefore address this by adding a statement in the Methods under sub-heading that the data were derived from the STRUETH study of twin pairs, and include a reference to the study. The paper can then be accepted for publication. Thank you for considering the Journal of Physiology to publish your research.

REFEREE COMMENTS

Referee #1:

The authors have sufficiently addressed my comments and suggestions

Referee #2:

The authors replied to my comments as to the differences between males and females at the start of the study in terms of FMD by referring to their previous study.

"We comprehensively assessed changes in CV risk factors to each form of exercise training and compared these in males and females. We found no evidence for differences between men and women in the changes observed in body composition, blood pressure, lipid profiles, blood glucose or insulin levels between males and females to either form of training. This makes it unlikely that changes in these factors explain the sex differences in FMD in the present study".

It means that the reader can not have an insight into the results without referring to the previous study, I cannot accept this as a valid response to the present paper. In other words, the results of the present paper can not be interpreted without going back to another paper published by authors.

The authors replied to my comment on high individual variation in the response to exercise in the following way:

Visual inspection of individual responses to training (i.e. Figure 1) supports wide a range of individual responsiveness to both forms of training. This is generally consistent with recent human studies of exercise responsiveness to other physiological variables.

I would rather think that the control of the exercise workload or intensity was not enough controlled or not adjusted to an individual and this could be also a reason. Otherwise such a huge variability as in this study, needs to have some biological explanation or an parameter identified behind ?

Referee #3:

The authors have responded satisfactorily to the Reviewers' previous concerns.

END OF COMMENTS

JP-RP-2024-287534 rebuttal

Impact of resistance and endurance exercise training on femoral artery function: sex differences in humans

Daniel J. Green[1], Hannah J. Thomas[1], Channa E. Marsh[1], Leanne Lester[1,2], Louise H. Naylor[1], Andrew Haynes[1]

[1]School of Human Sciences (Exercise and Sport Science), and [2]Business School (Centre for Social Impact), The University of Western Australia, Perth, Western Australia, Australia

Running title: Exercise modalities and femoral artery function

TOC Category: Cardiovascular

Author for correspondence:

Winthrop Professor Daniel J Green

School of Human Sciences (Exercise and Sport Science),

The University of Western Australia, Perth, WA, Australia, 6009

danny.green@uwa.edu.au, Phone +61 8 6488 2361

ORCID 0000-0003-3226-2921

Editor Comments:

Thank you for the revised manuscript which has been considered by the same reviewers that considered the original submission, and also a third reviewer due to a difference of opinion between Reviewer 1 and 2 regarding the revisions. I am happy to say that the majority opinion is that the paper should be accepted for publication in The Journal of Physiology. However, there is one further point that needs to be addressed please. The authors state in their response to the reviewers that "the data in this manuscript are indeed derived from our STRUETH study of twin pairs. Femoral FMD was only collected in a sub-sample of those participants. We have added information in the manuscript to clarify this point", but it appears that this information is not overtly clear in the manuscript. Please therefore address this by adding a statement in the Methods under sub-heading that the data were derived from the STRUETH study of twin pairs, and include a reference to the study. The paper can then be accepted for publication. Thank you for considering the Journal of Physiology to publish your research.

Response: The first sentence of the Methods under the heading *Participants* now reads:
Data presented in this paper relate to a cross-over exercise intervention study of twin pairs, referred to as the STRUETH study (Studied of Twin Responses to Understand Exercise as a Therapy) to Exercise involving 70 healthy but relatively inactive young adult participants (i.e. 35 twin-pairs) (see Supplementary Consort figure). All individuals completed brachial vascular measurements, data from which have been previously published (Green *et al.*, 2023). Femoral vascular data were collected in a subset of these participants, with data from 39 individuals (17 males and 22 females) included in this paper. This femoral measurement group was a convenience sample from the larger study, based on availability of trained sonographers to perform femoral vascular measurements whilst the brachial measures were simultaneously collected by another sonographer, and the tolerance of participants to additional testing.

Dear Professor Green,

Re: JP-RP-2025-287534R2 "Impact of resistance and endurance exercise training on femoral artery function: sex differences in humans" by Daniel J Green, Hannah J Thomas, Channa E Marsh, Leanne Lester, Louise Naylor, and Andrew Haynes

We are pleased to tell you that your paper has been accepted for publication in The Journal of Physiology.

The last Word (or similar) version of the manuscript provided will be used by the Production Editor to prepare your proof. When this is ready you will receive an email containing a link to Wiley's Online Proofing System. The proof should be thoroughly checked and corrected as promptly as possible.

Authors should note that it is too late at this point to offer corrections prior to proofreading. Major corrections at proof stage, such as changes to figures, will be referred to the Editors for approval before they can be incorporated. Only minor changes, such as to style and consistency, should be made at proof stage. Changes that need to be made after proof stage will usually require a formal correction notice.

All queries at proof stage should be sent to: TJP@wiley.com.

Following copyediting and typesetting the accepted version will be published online.

Yours sincerely,

Paul Greenhaff
Senior Editor
The Journal of Physiology

IMPORTANT POINTS TO NOTE FOLLOWING YOUR PAPER'S ACCEPTANCE:

If you would like to receive our 'Research Roundup', a monthly newsletter highlighting the cutting-edge research published in The Physiological Society's family of journals (The Journal of Physiology, Experimental Physiology, Physiological Reports, The Journal of Nutritional Physiology and The Journal of Precision Medicine: Health and Disease), please click this link, fill in your name and email address and select 'Research Roundup':
https://www.physoc.org/journals-and-media/membernews

• TRANSPARENT PEER REVIEW POLICY: To improve the transparency of its peer review process, The Journal of Physiology publishes online as supporting information the peer review history of all articles accepted for publication. Readers will have access to decision letters, including Editors' comments and referee reports, for each version of the manuscript as well as any author responses to peer review comments. Referees can decide whether or not they wish to be named on the peer review history document.

• You can help your research get the attention it deserves! Check out Wiley's free Promotion Guide for best-practice recommendations for promoting your work at: www.wileyauthors.com/eeo/guide. You can learn more about Wiley Editing Services which offers professional video, design, and writing services to create shareable video abstracts, infographics, conference posters, lay summaries, and research news stories for your research at: www.wileyauthors.com/eeo/promotion.

• IMPORTANT NOTICE ABOUT OPEN ACCESS: To assist authors whose funding agencies mandate public access to published research findings sooner than 12 months after publication, The Journal of Physiology allows authors to pay an Open Access (OA) fee to have their papers made freely available immediately on publication.

The Corresponding Author will receive an email from Wiley with details on how to register or log-in to Wiley Authors Services where you will be able to place an order.

• You can check if your funder or institution has a Wiley Open Access Account here: https://authorservices.wiley.com/author-resources/Journal-Authors/licensing-and-open-access/open-access/author-compliance-tool.html.

EDITOR COMMENTS

Senior Editor:

Comments to the Author:
Thank you for making this final amendment to the manuscript regarding the STRUETH study, which is now clear. I am

happy to say the paper is now acceptable for publication. Thank you for considering The Journal of Physiology for publication of your research.
